# Dietary curcumin restores insulin homeostasis in diet-induced obese aged mice

**DOI:** 10.18632/aging.203821

**Published:** 2022-01-11

**Authors:** Su-Jeong Lee, Prabha Chandrasekran, Caio Henrique Mazucanti, Jennifer F. O’Connell, Josephine M. Egan, Yoo Kim

**Affiliations:** 1 Department of Nutritional Sciences, Oklahoma State University, Stillwater, OK 74078, USA; 2 Laboratory of Clinical Investigation, National Institute on Aging (NIA), Baltimore, MD 21224, USA

**Keywords:** curcumin, type 2 diabetes, aging, insulin homeostasis, insulin-degrading enzyme (IDE)

## Abstract

Although aging is a physiological process to which all organisms are subject, the presence of obesity and type 2 diabetes accelerates biological aging. Recent studies have demonstrated the causal relationships between dietary interventions suppressing obesity and type 2 diabetes and delaying the onset of age-related endocrine changes. Curcumin, a natural antioxidant, has putative therapeutic properties such as improving insulin sensitivity in obese mice. However, how curcumin contributes to maintaining insulin homeostasis in aged organisms largely remains unclear. Thus, the objective of this study is to examine the pleiotropic effect of dietary curcumin on insulin homeostasis in a diet-induced obese (DIO) aged mouse model. Aged (18-20 months old) male mice given a high-fat high-sugar diet supplemented with 0.4% (w/w) curcumin (equivalent to 2 g/day for a 60 kg adult) displayed a different metabolic phenotype compared to mice given a high-fat high-sugar diet alone. Furthermore, curcumin supplementation altered hepatic gene expression profiling, especially insulin signaling and senescence pathways. We then mechanistically investigated how curcumin functions to fine-tune insulin sensitivity. We found that curcumin supplementation increased hepatic insulin-degrading enzyme (IDE) expression levels and preserved islet integrity, both outcomes that are beneficial to preserving good health with age. Our findings suggest that the multifaceted therapeutic potential of curcumin can be used as a protective agent for age-induced metabolic diseases.

## INTRODUCTION

The prevalence of diabetes in older adults has increased over the last few decades; almost 1 in 4 adults over age 65 in the United States are diagnosed as having diabetes [1]. Type 2 diabetes (T2D), the most prevalent type of diabetes in the elderly, is due to insulin resistance in insulin-sensitive tissues such as muscle, liver, and fat with the declining function of pancreatic β-cells in islets of Langerhans. The development of age-related insulin resistance and hyperglycemia is directly linked to obesity, which is a substantial risk for geriatric syndromes and medical complications such as non-alcoholic fatty liver disease causing hepatitis, fibrosis, cirrhosis, and cardiovascular disease [2–4]. Thus, finding a solution that leads to improved insulin sensitivity and fine-tuned insulin levels is critical for an aging society.

Maintaining normoglycemia can be achieved by improving two key components: β -cell functional integrity and hepatic insulin sensitivity [5]. Diminished insulin action in hepatocytes causes increased insulin synthesis and secretion, and expansion of β -cell mass as a compensatory response [6, 7]. Secreted insulin enters the liver through the portal circulation during which time over 50% of the insulin is cleared by the hepatocytes in the liver [8, 9]. A recent clinical study reported that reduced insulin clearance due to reduced insulin-degrading enzyme (IDE) activity impacting recycling of insulin receptors back to plasma membrane contributes to increased insulin secretion into the portal vein, thereby further desensitizing hepatocytes to insulin [10].

Curcumin is a bioactive polyphenolic compound extracted from the herb *Curcuma longa* [11]*.* It has antioxidant, anti-inflammatory, and anti-diabetic properties which should mitigate age-associated diseases [11–13]. Its basic mechanism of action as an anti-diabetic therapeutic agent was largely unknown [13–18]. However, we previously demonstrated that curcumin supplementation increases insulin sensitivity in hepatocytes by upregulating hepatic IDE expression and preserved islet integrity in a diet-induced obese (DIO) mouse model. Furthermore, we enumerated various cellular and molecular events in the insulin signaling pathway regulated by curcumin [19]. In this study, we sought to determine if curcumin supplementation during a nutrient stressor known to cause insulin resistance in aged mice might be beneficial to preserving hepatocytes in an insulin-sensitive phenotype while protecting β -cells from the need to compensate for insulin resistance.

## RESULTS

### Curcumin supplementation suppresses body weight gain and fat accumulation in aged mice

**Figure 1:**
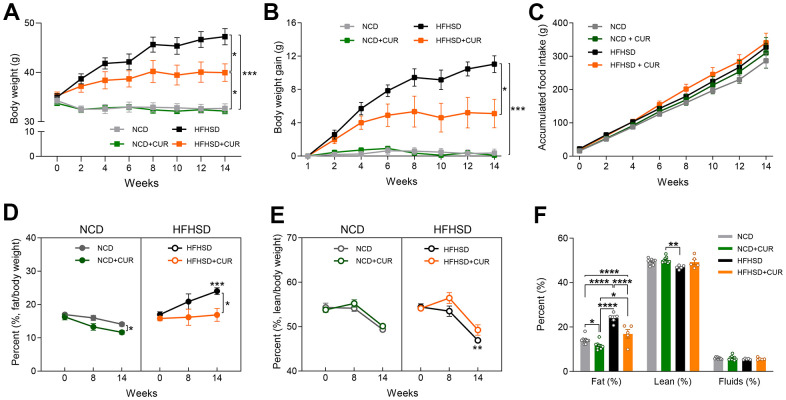
**Curcumin ameliorates obesity in high-fat high-sugar diet (HFHSD) induced obese aged mice.** (**A**) body weight (g), (**B**) body weight gain (g), (**C**) accumulated food intake (g) (n = 7-9). NMR spectrometry was conducted with normal chow diet (NCD), curcumin-supplemented normal control diet (NCD+CUR), high-fat high-sugar diet (HFHSD), and curcumin supplemented high-fat high-sugar diet (HFHSD+CUR) fed mice at baseline, 8 weeks, and 14 weeks of diet (n = 5-9). (**D**) fat/body weight percentage (%) (**E**) lean/body weight percentage (%) (**F**) overall comparison of fat/body weight percentage (%), lean/body weight percentage (%) and fluid/body weight percentage (%) at week 14. *p ≤ 0.05, **p ≤ 0.01, ***p ≤ 0.001 and ****p ≤ 0.0001.

For this study, we used 18-20 months old male C57BL/6 mice (n = 7-9 in each group) and administered a dietary intervention for 15 weeks. We used two dietary regimes in four groups of mice: normal chow diet (NCD) with or without curcumin (CUR) supplementation and high-fat high-sugar diet (HFHSD) with or without CUR. HFHSD+CUR fed mice had markedly lower body weights (39.57 ± 1.42 g vs. 43.67 ± 2.01 g) and less body weight gain (4.72 ± 1.78 g vs. 7.83 ± 1.64 g) compared to HFHSD fed mice (Figure 1A, 1B), without any change in food intake (Figure 1C). On the other hand, NCD and NCD+CUR fed mice maintained similar body weights and food intake (Figure 1A–1C).

Because of the weight loss due to CUR supplementation, we analyzed body composition using NMR. As we expected, HFHSD significantly increased fat/body percentage (%) compared to NCD for 14 weeks (p = 0.0003). CUR fed mice had lower total body fat mass in both dietary regimes from week 8 onwards and prevented body fat accumulation in the HFHS group such that their fat mass was similar to NCD mice, despite no alteration in their food intake (16.88 ± 1.92% vs 24.02 ± 1.00%, Figure 1D, right). As a gradual decline of lean body mass is known to occur with aging in mice [20], we also monitored lean body mass. We observed a significant loss of lean body mass in the HFHSD group compared to NCD by week 14 (p = 0.0056). A protective effect of CUR on the loss of lean body mass was observed in both dietary regimes compared to the respective controls by week 8 (mouse age, 20-22 months old). However, this effect was not maintained by the end of the study (Figure 1E). In addition, we analyzed overall fat and lean body mass (%) and fluids (%) at week 14 and found that only fat mass was reduced by CUR (Figure 1F).

### Curcumin changes hepatic gene expression profiling in aged mice

**Figure 2:**
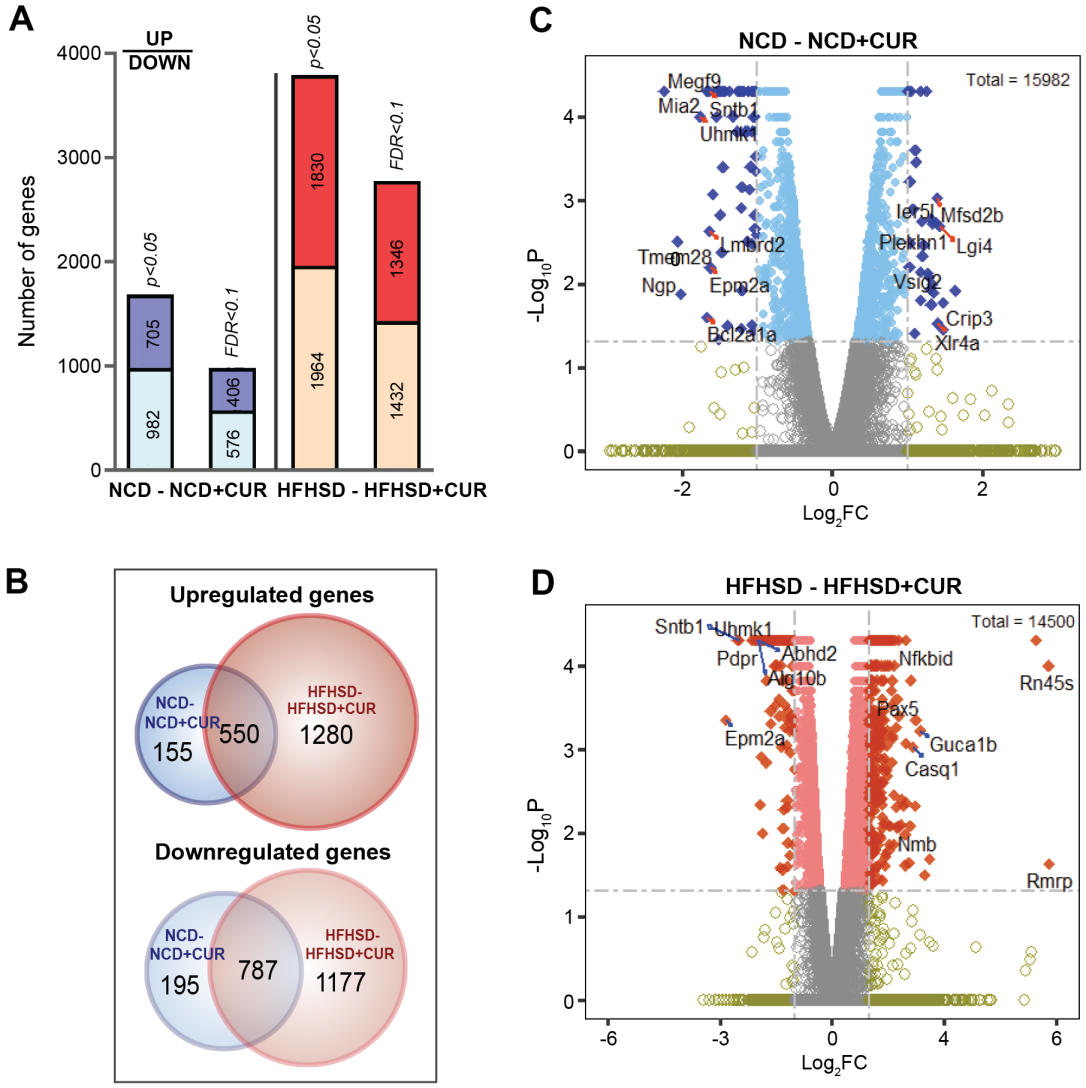
**Liver RNA sequencing transcriptome profiling in NCD, NCD+CUR, HFHSD, and HFHSD+CUR fed aged mice.** (**A**) Number of differentially regulated genes (DEGs) as defined by p-value and False Discovery Rate (FDR)-defined cut-offs are presented for comparisons of mice groups that received curcumin-supplemented normal control diet (NCD+CUR) and high-fat, high-sugar diet (HFHSD+CUR) to their non-supplemented NCD/HFHSD counterparts (n = 3 per group). The bottom part represents downregulated genes and the top part, upregulated genes. (**B**) Venn Diagrams represent overlapping and distinct gene expression between the comparison groups for upregulated (top) and downregulated (down) genes. (**C**, **D**) The number of genes with ≥ 2 log_2_ fold change (FC; represented as dark blue or dark orange) and p-value < 0.05 are indicated in volcano plots for two different comparisons.

To characterize any CUR-driven transcriptional changes in the liver of aged mice, we performed whole transcriptome RNA sequencing (RNAseq) analysis. The analysis generated 40,785,830 to 71,480,323 reads, which represented a 90-98% mapping rate to the reference genome (Supplementary Table 1). As shown in Figure 2A, CUR altered hepatic gene expression compared to non-CUR mice. The numbers of upregulated and downregulated genes are presented for data analysis using p-value cut-off < 0.05 and a false discovery rate (FDR) cut-off < 0.1. To capture all the true positives, a p-value cut-off was utilized to define the differentially expressed genes (DEGs). There were 1687 and 3794 genes that showed a significant change with CUR in NCD and HFHSD groups compared to their respective non-CUR groups (Figure 2A). There were at least 8-fold higher uniquely upregulated and 6-fold uniquely downregulated genes in the HFHSD+CUR group when compared with their NCD counterparts (Figure 2B). The distribution of the log_2_FC (fold change) and p-values with selected upregulated and downregulated genes are presented in the volcano plots (Figure 2C, 2D). Genes with ≥ 2-fold change of gene expression are shown in dark blue for the NCD group (32 up- and 62 down-regulated genes) and dark orange for the HFHSD group (382 up- and 186 down-regulated genes). Taken together, the DEG data indicate that CUR altered the liver transcriptome in the aged mice and altered more genes in the HFHSD groups.

### Dietary curcumin alters gene expression associated with insulin signaling and senescence pathways in the aged mice

**Figure 3:**
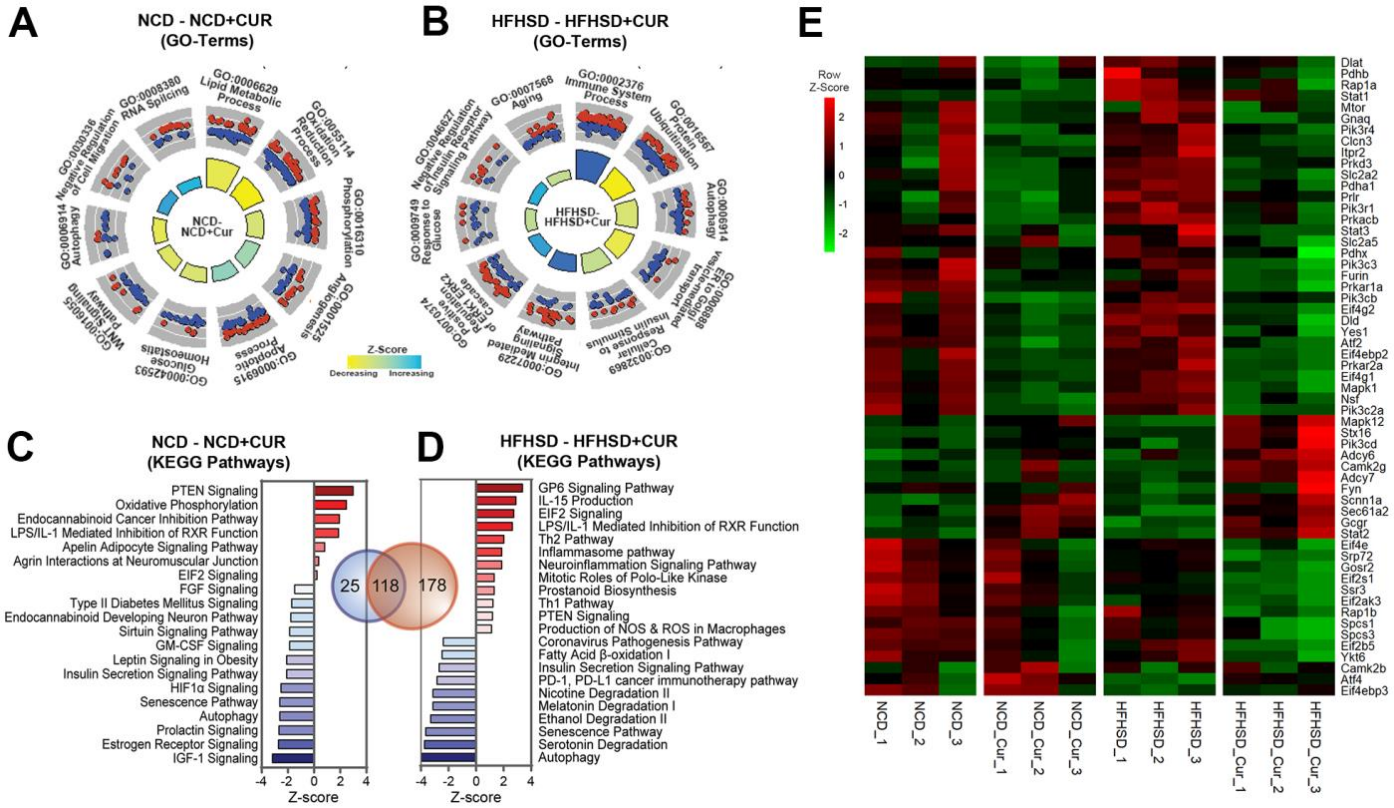
**RNA sequencing analysis of the liver reveals that curcumin supplementation regulates a broad array of genes related to aging and insulin homeostasis.** (**A,** **B**) Significant Gene Ontology (GO)-Biological Processes enriched in Differentially Expressed Genes (DEGs) in aged mice liver receiving curcumin-supplemented normal control diet (NCD+CUR) or high-fat, high-sugar diet (HFHSD+CUR) compared with their respective non-supplemented control groups (NCD or HFHSD) (n = 3 per group). The relevant top 10 GO-terms are presented in the GO Circle plots. Within each GO-Term, the upregulated and downregulated genes are represented in red and blue circles. The breadth of inner rectangles represents the strength of p-value significance. Yellow color represents GO-Terms with negative Z-scores and blue, positive Z-scores. (**C**, **D**) Bar charts of top significantly affected canonical pathways based on IPA presented based on the Z-scores. The red color indicates activation, and the blue color indicates suppression. (**E**) Heatmap analysis of insulin signaling pathway: Heatmap of RNA expression is measured by FPKM (p < 0.05 in HFHSD comparison; FPKM > 1.5) from Insulin Receptor and Insulin Secretion signaling pathways. Red indicates a positive Z-Score and green, negative Z-score.

To investigate the biological significance of the differentially regulated genes, GO (Gene Ontology) terms and canonical pathways associated with DEGs were identified. The most significant enrichments, especially in the HFHSD+CUR group, were for (a) glucose metabolism- and homeostasis-related pathways, (b) aging and aging-related pathways, and (c) pathways that control inflammation such as Th1, Th2, inflammasome, PD-1/PD-L1 pathways (Figure 3A, 3B). The notable canonical pathways shared between both CUR groups in the different dietary regimens included insulin secretion and insulin receptor signaling pathways, the senescence pathway, IGF-1, mTOR, and Hif1 α signaling, and Type 2 diabetes mellitus signaling (Figure 3C). Heatmap analysis for genes in the insulin signaling pathway revealed that CUR in both dietary regimes led to the downregulation of genes that attenuate insulin signaling transduction (Figure 3E). This response was more evident in the HFHSD groups. Analysis of upstream transcription regulators (TRs) associated with DEGs in HFHSD+CUR compared to HFHSD groups identified 187 significant TRs of which 15 had positive Z-scores associated with activation and 16 with inhibition (Supplementary Figure 1). The genes regulated by hepatic nuclear factors, Hnf1A (n = 112 target molecules in the dataset) and Hnf4A (n = 527) TRs, whose mutations are known to cause maturity-onset diabetes of the young (MODY) and insulin-independent diabetes mellitus [21, 22], were predicted to be inhibited by CUR. The activation Z-scores for Hnf1A and Hnf4A were -4.749 and -3.314, respectively. Thus, the RNAseq data suggested that CUR prevents reduction in insulin sensitivity in the liver in HFHSD-fed aged mice through transcriptional regulation of insulin- and aging-related pathways.

### Curcumin treatment maintains insulin homeostasis during aging and dietary challenge that is mediated by hepatic IDE

**Figure 4:**
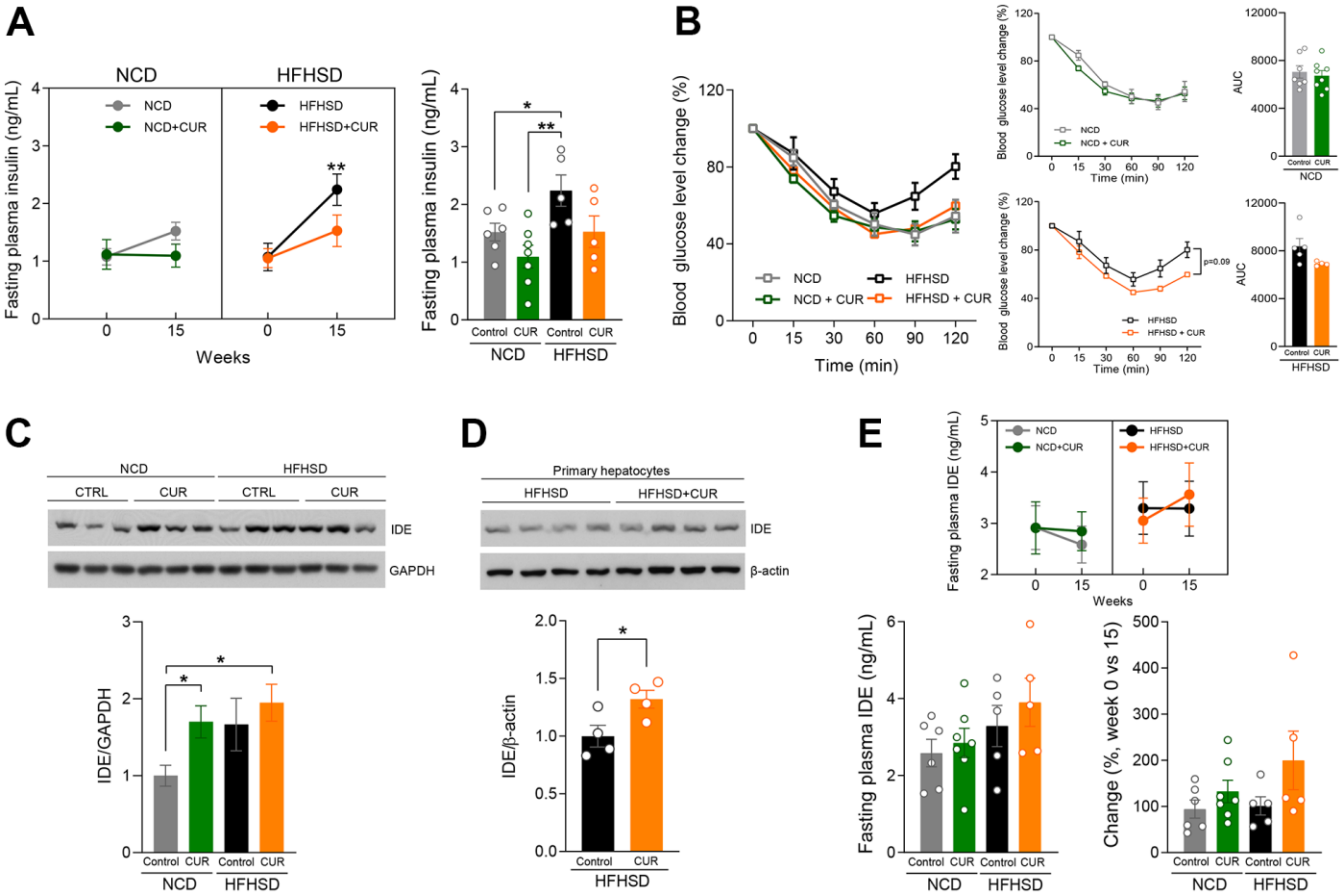
**Curcumin supplementation raised insulin sensitivity by IDE expression.** (**A**) 6-hours fasting insulin levels (ng/mL) at week 15 (n = 5-7). (**B**) Insulin Tolerance Test (ITT) was performed for 14 weeks after diet treatment, and the area under the curve (AUC) analysis (n = 4-8). (**C**) IDE and GAPDH protein expression levels in liver tissue lysate 15 weeks after diet treatment (n=3) (**D**) IDE and β-actin protein expression levels in primary hepatocytes 15 weeks after diet treatment (n=4) (**E**) fasting plasma IDE (ng/mL) basal level and 15 weeks after diet treatment (n = 5-7). *p ≤ 0.05 and **p ≤ 0.01.

Considering that CUR supplementation altered hepatic genes in the insulin signaling pathway, we then explored this pathway more closely. Fasting plasma insulin (FPI) did not change over time in the NCD+CUR group while in the NCD-fed group had almost a 50% increase by week 15 (1.10 ± 0.53 vs. 1.52 ± 0.38 ng/mL: p = 0.058). While HFHSD, as expected, caused a highly significant increase in FPI by 15 weeks compared to baseline (p < 0.01), in the HFHSD+CUR group this was much less (1.08 ± 0.49 vs. 1.53 ± 0.61 ng/mL; p = 0.14) than with HFHSD alone and was similar to that of the NCD group at that time (Figure 4A). During an insulin tolerance test (ITT; 1 U insulin/kg body weight) HFHSD+CUR group had similar insulin sensitivity to NCD and NCD+CUR fed groups, whereas the HFHSD fed group, again as expected, had reduced insulin sensitivity (Figure 4B). These *in vivo* results lend credence to the finding that CUR positively modulates insulin sensitivity. Our previous study uncovered that oral CUR supplementation regulates hepatic IDE protein levels [19]. To examine if this effect is also seen in aging mice, we conducted immunoblotting and analyzed IDE protein expression levels in the liver of all four groups. We found that NCD+CUR fed mice had 1.7-fold more IDE protein levels than NCD fed mice. Although HFHSD+CUR mice had slightly increased IDE expression levels compared to HFHSD alone, it did not reach a significant difference (Figure 4C). However, as insulin clearance occurs in all cells in addition to hepatocytes [23], we isolated primary hepatocytes from mice fed HFHSD or HFHSD+CUR for 15 weeks. CUR supplemented mice had significantly higher IDE protein levels compared to HFHSD alone (Figure 4D). Since our results have shown that CUR increased IDE levels in hepatocytes, we then examined circulating IDE levels after 6 hours of fasting and we found that only HFHSD+CUR fed mice displayed a trend of increased fasting plasma IDE levels at week 15 compared to week 0 (Figure 4E).

### Curcumin supplementation preserves pancreatic islet integrity in the aged mice

**Figure 5:**
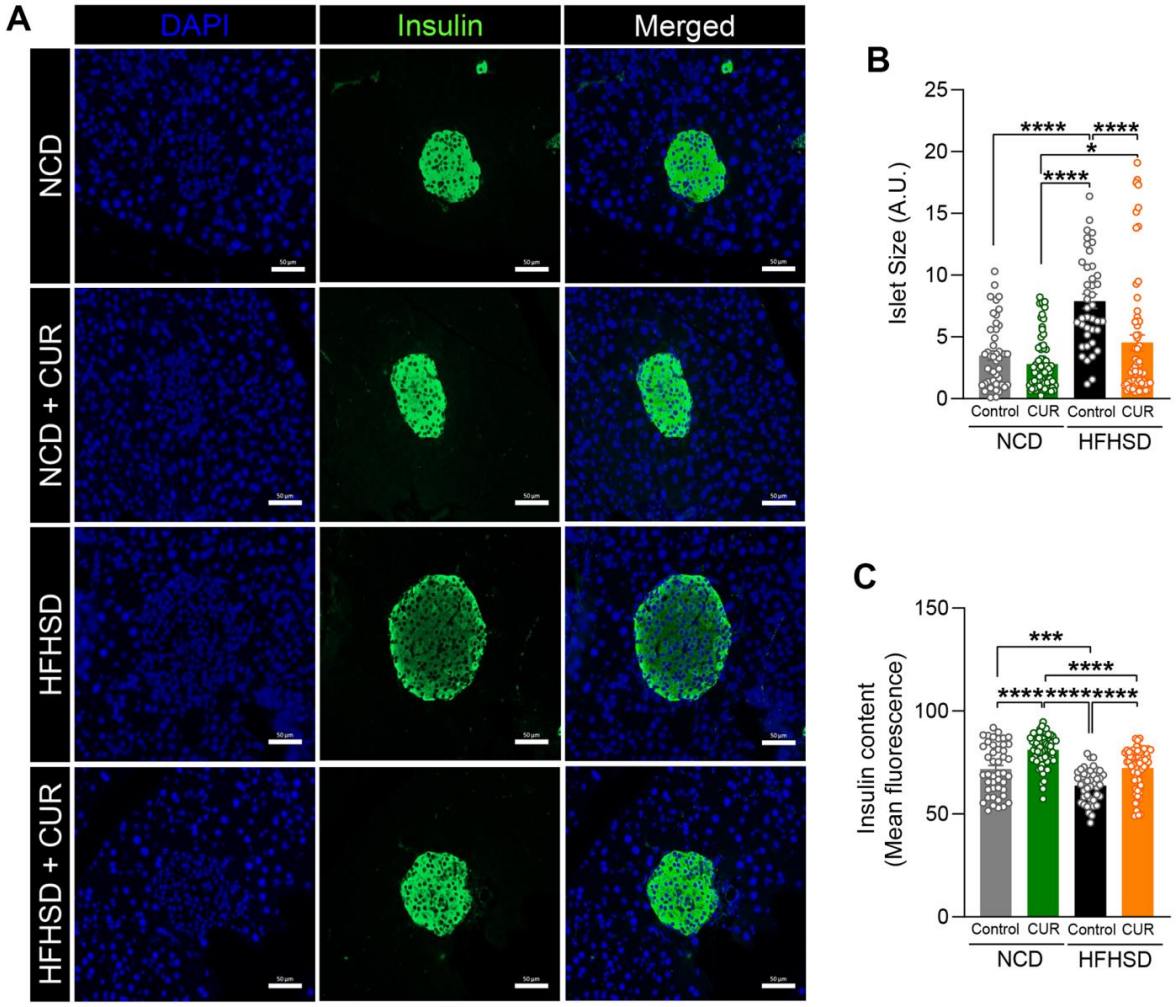
**HFHSD+CUR fed mice maintain normal pancreatic beta-cell integrity.** After 15 weeks of diet, (**A**) pancreas sections from mice on normal chow diet (NCD), curcumin-supplemented normal control diet (NCD+CUR), high-fat high-sugar diet (HFHSD), and curcumin supplemented high-fat high-sugar diet (HFHSD+CUR) were stained for insulin (green) and DAPI (blue). Representative confocal images are shown using a 40X oil objective. Fiji-Image J software (NIH) was used to quantitate (**B**) total islet size (scale bar, 50 μm), (**C**) insulin content (mean fluorescence). Quantitative analysis was based on at least 50-70 images per group (n = 3 mice per group). *p ≤ 0.05, ***p ≤ 0.001 and ****p ≤ 0.0001.

The above findings indicate that dietary curcumin prevents hyperinsulinemia in the HFHSD group because of enhanced insulin sensitivity in the liver, and therefore should result in alleviating the need for the increased demand for insulin secretion from β-cells. We therefore examined islet integrity using insulin immunofluorescence staining of fixed pancreata after 15 weeks of the diets in the four groups (Figure 5A). The average islet size with HFHSD was significantly larger (7.89 ± 0.58 A.U) than with NCD (3.48 ± 0.39 A.U; p < 0.0005), as expected. While CUR supplementation did not alter islet size during NCD, in the HFHSD+CUR group, the average islet size was much smaller than that of the HFHSD alone group (3.48 ± 0.39 A.U vs. 2.80 ± 0.27 A.U; p < 0.0001) (Figure 5B). Along with an examination of islet morphology, we quantified the intensity of labeled insulin. CUR supplementation resulted in significantly increased insulin content in islets in both NCD and HFHSD groups. The insulin content in the HFHSD+CUR group was no different from NCD and NCD+CUR groups (Figure 5C).

## DISCUSSION

Our previous work found that dietary curcumin improves insulin clearance and preserves islet integrity in middle-aged obese mice [19]. However, any effect of dietary curcumin on the age-associated loss of insulin sensitivity was not previously studied. Consistent with our previous results in the middle-aged mice, curcumin supplementation prevented body weight gain under obesity-inducing conditions while food intake was not altered. In hepatocytes, curcumin improved insulin sensitivity and increased hepatic IDE protein levels. It has been reported that patients with T2D have reduced hepatic IDE expression levels accompanied by lower insulin clearance [24–26]. Furthermore, a recent study found that hepatic IDE-overexpression in DIO mice improves insulin sensitivity and glucose tolerance that is compensated for by reduced plasma insulin levels. Conversely, liver-specific IDE knockout (L-IDE-KO) mice fed a high-fat diet (HFD) showed insulin resistance and glucose intolerance accompanied by elevated fasting insulin levels [27, 28]. These studies confirm the important role of the degree of insulin signaling in hepatocytes to feedback to insulin secretion. Thus, elevated liver-specific IDE level orchestrates the concentration of insulin in peripheral tissues [29]. In our study, increased hepatic insulin sensitivity due to curcumin’s beneficial effect on IDE was reflected in smaller islets and increased insulin content, illustrating less demand for insulin secretion and synthesis. Therefore, we conclude that curcumin has beneficial effects in preserving insulin sensitivity in the liver, even in very old mice, and even when they are subjected to severe adverse dietary conditions.

The global liver transcriptomic RNAseq analyses confirm the beneficial effects of dietary curcumin on insulin signaling-related gene expression, even in aged mice livers. Although the curcumin-induced transcriptional response was different between aged mice on a normal chow diet and nutritionally challenged mice, curcumin supplementation upregulated and downregulated genes (550 and 787 genes, respectively) in both groups. Moreover, the GO enrichment analysis identified insulin receptor binding (GO:0005158; p = 0.019) and the KEGG pathway analysis identified the insulin resistance pathway as the most significant pathway associated with the commonly regulated genes (p = 7. 31E^-0.4^; fold enrichment = 2.497). It lends further weight to the finding that curcumin supplementation indeed had a beneficial effect on insulin signaling even in aged mice. Additionally, and again of relevance to aging, among the top 10 pathways, the senescence pathway, was notably suppressed (Z-score = -3.679, HFHSD+CUR vs HFHSD), indicating the potential benefits of curcumin supplementation as an anti-aging agent.

Interestingly, the autophagy pathway was also influenced by curcumin supplementation of both NCD and HFHSD based on GO-term and pathway enrichment analysis. Autophagy and the insulin signaling pathway are inextricably linked [30, 31]. Recent studies have demonstrated that upregulating autophagy genes lead to amelioration of insulin resistance and enhancement of insulin sensitivity in obese mice [32–34]. Although prior investigations uncovered the relationship between IDE and secretory autophagy in the brain [35–37], in the case of hepatocytes, insulin sensitivity and downstream signaling play a role in autophagy. For example, low circulating insulin levels drive increased autophagic flux, contributing to the maintenance of normal blood glucose levels [38]. Thus, crosstalk between hepatic IDE and autophagy that is modulated by curcumin supplementation is involved in hepatocyte response to circulating insulin levels.

Besides dietary manipulations to improve insulin signaling in hepatocytes by reducing body weight, current pharmacological therapies to improve hepatic insulin sensitivity include thiazolidinediones (TZDs) and metformin. However, while TZDs improve insulin sensitivity and reduce lipid accumulation in liver [39], they result in weight gain and fluid retention over time [40] while metformin had not been shown to have any benefits in people over the age of 65 [41]. We now report that not only has dietary curcumin beneficial effects on insulin in action in liver with age, but that this is reflected in protection from increasing demand for insulin secretion from ß-cells. Therefore, the intake of curcumin, a safe and effective dietary supplement, in a daily regimen may be an ideal way to preserve hepatocyte and ß-cell health with age.

There are some limitations in this study. Considering that dietary curcumin has low aqueous solubility, and poor oral bioavailability [42], supplementation of curcumin by incorporating it into diets might result in less biological efficacy in preventing age-associated declines in insulin downstream signaling. However, in the current study, we focused on the effect of dietary curcumin on IDE expression levels in the liver and even under these limitations, we still found significant effects with incorporation of curcumin directly into the diets. Other curcumin formulations might have resulted in even greater beneficial effects. Additionally, we have not yet studied if these effects now herein documented are specific to hepatocytes or if they occur in other IDE-containing tissues, such as brain, where reduced insulin action is well-described from epidemiological studies to be a feature of cognitive decline with age. We are going to undertake such studies.

In summary, our study illustrates that curcumin supplementation in aged individuals is likely to play a role in mitigating reduced hepatic insulin sensitivity due to aging *per se* and dietary challenges. It seems convincing that curcumin positively regulates insulin sensitivity in hepatocytes because of the upregulation of IDE that is reflected in reduced demand for increased insulin secretion and synthesis. Thus, curcumin is a potent, natural therapeutic agent which acts in a multifaceted manner to protect aging-induced metabolic disorders.

## MATERIALS AND METHODS

### Animals and treatment

All animal procedures were performed in an American Association for Accreditation of Laboratory Animal Care accredited facility and the procedures were approved by the Animal Care and Use Committee of the NIA Intramural Program. Aged male C57BL/6 mice (18-20 months old) were obtained from the National Institute on Aging (NIA) Aged Rodent Colony housed at Charles River Laboratories (Frederick, MD). After being transferred into the NIA intramural housing facility (Baltimore, MD), animals were acclimated to the facility for 1 week with standard NIH chow (Teklad Global Rodent Diet, Envigo, Indianapolis, IN). Animals were subject to baseline assessments such as body weight and body composition, then blood samples were collected for analyses of plasma markers. After baseline assessment, they were randomized into four groups: a normal chow diet (NCD; n = 9), normal chow diet containing 0.4% (w/w) of curcumin (NCD+CUR; n = 9) a high fat/high sugar diet (HFHSD; n = 8) or a HFHSD containing 0.4% (w/w) of curcumin (HFHSD+CUR; n = 7) for 15 weeks. Curcumin for diets was purchased from Sigma-Aldrich (St. Louis, MO) and all the diets were formulated by Dyets Inc. (Bethlehem, PA). The dose of curcumin in this study was determined based on our previous study using middle-aged mice. This amount of curcumin is equivalent to 2 g/day for a 60 kg adult calculated with an equivalent surface area dosage conversion method [43]. Mice were allowed *ad libitum* access to food and water throughout the study. The average body weight and food consumption were calculated weekly for 15 weeks.

### Insulin tolerance test

For insulin tolerance test (ITT), blood glucose levels were measured from tail veins of 6 hours fasted mice at 0, 15, 30, 60, 90, and 120 minutes following intraperitoneal administration of recombinant human insulin from Novo Nordisk (1 U insulin/kg body weight). Blood glucose levels were measured using a hand-held glucometer (Contour, Bayer, model 7160-P). Blood plasma was used to measure insulin levels using an Ultra-Sensitive Mouse Insulin ELISA kit (Crystal Chemical Inc., Downers Grove, IL).

### Immunoblotting analysis

Mouse liver tissue samples were homogenized in tissue lysis buffer (25 mM Tris (pH 7.4), 2 mM Na_3_VO_4_, 10 mM NaF, 10 mM Na_4_P_2_O_7_, 1 mM EGTA, 1 mM EDTA, and 1% NP-40) containing phosphatase and protease inhibitor cocktails in the OMNI BeadRuptor 24 (Omni-Inc, Kennesaw, GA). Protein was quantified using a BCA Assay (ThermoFisher Scientific, Rockford, IL) and then protein loading samples were resolved in SDS-PAGE under reducing conditions and transferred to polyvinylidene fluoride (PVDF) membrane. Membranes were blocked in blocking reagent (LI-COR, Lincoln, NE) assay system for 1 hour at room temperature and incubated with primary antibodies overnight at 4° C as follows: IDE (1:1000) from ThermoFisher Scientific (Waltham, MA) and GAPDH (1:3000) and β-actin (1:3000) from Cell Signaling Technology (Danvers, MA). Membranes were washed with tris-based saline-tween 20 (TBS-T) and the appropriate secondary antibody (1:20000, ThermoScientific, Rockford, IL) was added in blocking reagent for 1 hour at room temperature. Membranes were washed three times with TBS-T and developed using a chemiluminescence assay system. Bands on the membrane were visualized on autoradiography film. Western blot images were scanned, saved as Tiff files, inverted, and integrated density was analyzed using ImageJ software (NIH). Phosphorylated protein levels were normalized to the respective total protein levels.

### Assessment of body composition using NMR spectroscopy

We measured mouse body composition at week 0, 8, and 14. Mice were placed on a Bruker Minispec LF90 NMR (Bruker, Billerica, MA). Readouts were lean, fat, and fluid mass. Data are presented as percent body fat and lean masses.

### RNA and mRNA library preparation

Total RNA was extracted from the left lateral lobe of the liver. RNA purity was determined by using a NanoDrop8000 spectrophotometer. RNA integrity was assessed using an Agilent Technologies 2100 Bioanalyzer with an RNA Integrity Number (RIN) value. The mRNA sequencing libraries were prepared from 1 μg of RNA, according to the manufacturer’s instructions (Illumina Truseq stranded mRNA library prep kit). The quality of the amplified libraries was verified by capillary electrophoresis (Bioanalyzer, Agilent). The index tagged libraries were pooled on equimolar quantities, post to the quantitative real-time PCR using SYBR Green PCR Master Mix (Applied Biosystems). The sequencing was performed on Novaseq 6000 sequencing system (Illumina) with 2 x 100bp read length. The rate at which the required readings for each sample are mapped to the reference genome is presented in Supplementary Table 1.

### RNA sequencing analysis

The sequencing reads for each sample were mapped to the reference mm10 genome of Mus musculus (C57BL/6J strain) by Tophat (v2.0.13). The aligned results were added to Cuffdiff, a program of Cufflinks (v2.2.0) to identify differentially expressed genes (DEGs) using the UCSC genome annotations and default parameters. For library normalization and dispersion estimation, pooled methods were applied. Raw and processed data have been deposited to NCBI with GEO accession numbers (GSE186971). Genes exhibiting p value < 0.05 were considered significant. The sequencing and initial data analysis were performed by the DNA LINK sequencing lab (Los Angeles, CA).

### Gene ontology and pathway analysis

The differentially expressed genes (DEGs) were classified into Gene Ontology terms (GO-Term) through enrichment using DAVID (Database for Annotation, Visualization, and Integrated Discovery) online tool (https://david.ncifcrf.gov/home.jsp). The terms were categorized into GO-Molecular Function (MF), GO-Biological Process (BP), and GO-Cellular Component (CC). Functional pathway analysis was performed using Ingenuity Pathway Analysis (IPA, QIAGEN Inc., https://www.qiagenbioinformatics.com/products/ingenuity-pathway-analysis). The right-tailed Fisher’s exact test p-values were used to identify significant GO-Terms and pathways. The gene list associated with specific pathways was obtained from the KEGG database (https://www.genome.jp/kegg/pathway.html) and used to construct the heatmaps shown.

### Data analysis and visualization

Data analysis was done using JMP-SAS, MS-Excel, GraphPad prism, and R Studio 1.4.1106 (http://www.R-project.org). Volcano plots were generated using the EnhancedVolcano package of R. To better visualize and explore the enriched GO-Terms, the Gene Ontology obtained from the DAVID database and the expression data were combined and represented as GOCircle plots using the Goplot R package. The R package Dplyr was used for data processing and ggplot package was used to construct graphs. Heatmaps were constructed using pheatmap library. The gene list for signaling pathways was obtained from IPA and KEGG pathway databases. The figures were assembled in Adobe Illustrator and Adobe Photoshop.

### Immunofluorescence staining

At the end of the study, pancreata from NCD, NCD+CUR, HFHSD, and HFHSD+CUR fed mice were fixed in 4% paraformaldehyde overnight, embedded in paraffin blocks, and sectioned (5μm). After deparaffinization, heat-induced epitope retrieval of tissue sections was achieved using citrate buffer antigen retriever (Sigma), then washed, permeabilized, blocked, and insulin antibody was added overnight and stored at 4° C (1:100; Dako, Carpinteria, CA). Sections were incubated with fluorescently labeled secondary antibodies (1:1000; Alexa Fluor 488 or Alexa Fluor 594, ThermoFisher Scientific) and DAPI (1:5000; ThermoFisher Scientific) for nuclear staining after the washing procedure. Images were obtained on a Zeiss Axio Observer D1 / 5 Inverted Microscope in a 20x/0.8 objective. Islet size and insulin content were measured using Fiji-Image J software (NIH). Insulin content was determined as the mean fluorescence intensity of the green channel (insulin staining) within each islet. Quantitative analysis was based on at least 50-70 images per group (n = 3 per group).

### Statistical analysis

All data were analyzed by GraphPad Prism (Prism 9; GraphPad Inc.). Ordinary one-way ANOVA was held for fasting plasma insulin and IDE levels, IDE protein expression levels in the liver, pancreas islet size, and pancreas insulin content. Two-way ANOVA repeated measure was held for body weight, food intake, body composition, and ITT followed by Tukey’s multiple comparisons test; NMR followed by Šídák's multiple comparisons test. Student t-test held for IDE protein expression level in primary hepatocytes. Quantitative data are represented as the mean ± SEM. Quantification analysis for AUC, western blot band density, and imaging pixels was conducted using one-way ANOVA followed by Tuckey’s multiple comparisons after the outlier test (α=0.05). * p≤0.05, ** p≤0.01, ***p≤0.001, and **** p≤0.0001 were considered statistically significant.

## Supplementary Material

Supplementary Figure 1

Supplementary Table 1
