# Supplementary material for: Dietary curcumin restores insulin homeostasis in diet-induced obese aged mice
Source: Aging (Albany NY). 2022 Jan 11;14(1):225–39. doi: 10.18632/aging.203821 (PMC8791219; doi:10.18632/aging.203821)
Supplement: Supplementary Figure 1 [file aging-14-203821-s001.pdf]

SUPPLEMENTARY FIGURE

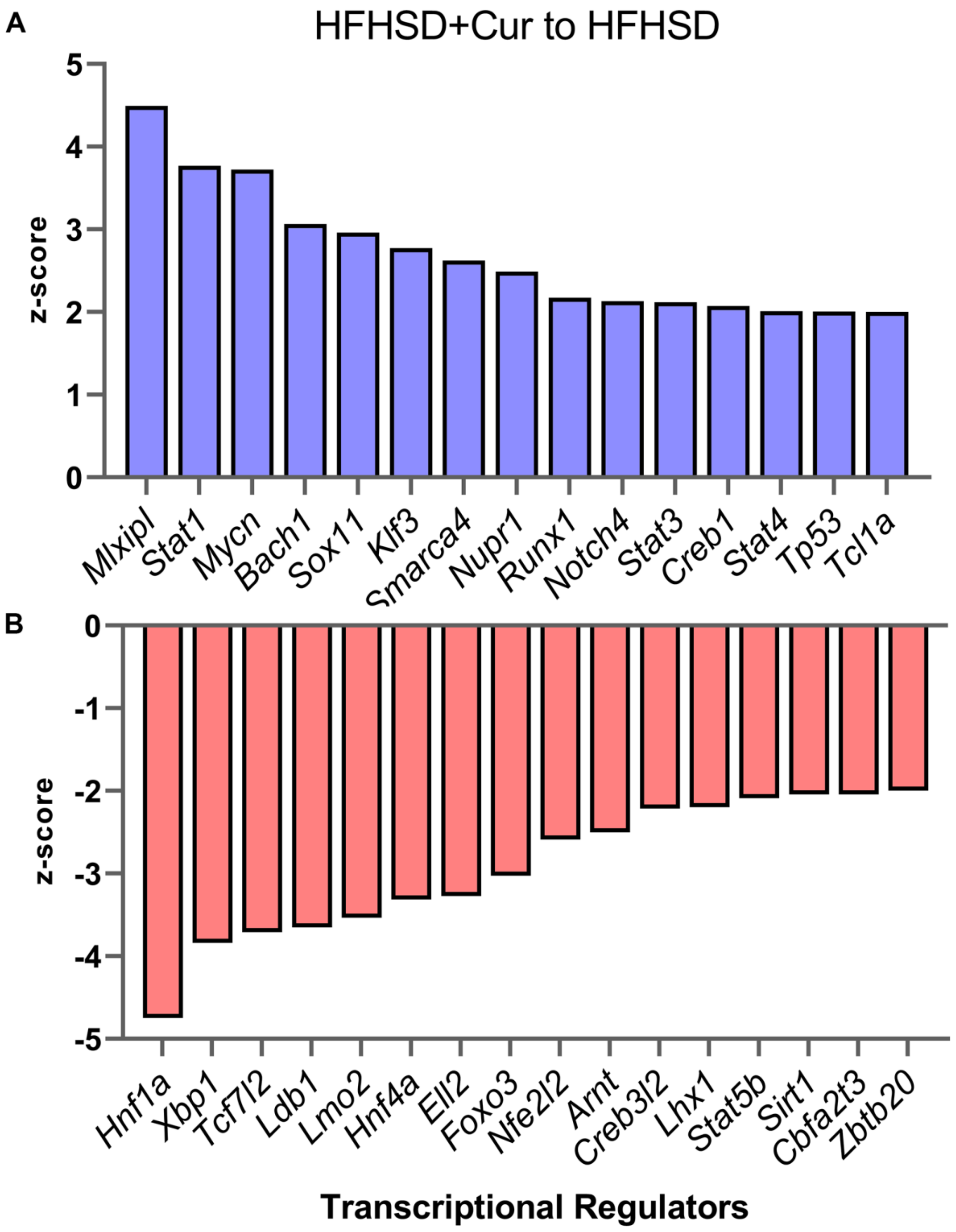

Supplementary Figure 1. Transcriptional regulators associated with differentially expressed genes. The z-scores as predicted by Ingenuity Pathway Analysis for upstream transcriptional regulators (TRs) associated with differentially expressed genes are shown for those predicted to be associated with activation (**A**) or inhibition (**B**) of downstream genes. The comparison is provided for aging mice fed with HFHSD+CUR and non-supplemented HFHSD control group.
