# Supplementary material for: Dietary curcumin restores insulin homeostasis in diet-induced obese aged mice
Source: Aging (Albany NY). 2022 Jan 11;14(1):225–39. doi: 10.18632/aging.203821 (PMC8791219; doi:10.18632/aging.203821)
Supplement: Supplementary Table 1 [file aging-14-203821-s002.pdf]

SUPPLEMENTARY TABLE

Supplementary Table 1. Alignment summary.

Sample	Reads	Mapped_reads	Mapping_rate
HFHSD_1	44,712,286	42,287,128	94.5761
HFHSD_2	55,922,916	53,161,527	95.0622
HFHSD_3	72,968,900	71,480,323	97.9600
HFHSD_Cur_1	56,314,214	51,156,887	90.8419
HFHSD_Cur_2	47,353,264	45,271,212	95.6031
HFHSD_Cur_3	53,605,040	51,339,781	95.7742
NCD_1	46,635,284	43,886,507	94.1058
NCD_2	49,468,774	46,352,953	93.7014
NCD_3	72,347,684	70,571,108	97.5444
NCD_Cur_1	48,303,724	45,757,951	94.7297
NCD_Cur_2	43,365,404	40,785,830	94.0515
NCD_Cur_3	45,002,994	42,087,349	93.5212
Average	53,000,040	50,344,880	94.7900
